# Public Attitudes and Predictors of Public Awareness of Personal Digital Health Data Sharing for Research: Cross-Sectional Study in Japan

**DOI:** 10.2196/64192

**Published:** 2025-10-09

**Authors:** Yasue Fukuda, Koji Fukuda

**Affiliations:** 1 Department of Pharmacy, Suzuka University of Medical Science, 3500-3 Minami-tamagaki, Suzuka, Mie, 513-8760, Japan, 81 59-373-7030, 81 59-368-1271; 2 Department of Political Science and Economics, Faculty of Political Science and Economics, Waseda University, Shinjuku-ku, Tokyo, Japan

**Keywords:** digital health data, cross-sectional study, predict factor, Japan, willingness to share personal data, eHealth literacy

## Abstract

**Background:**

As digital technology advances, health-related data can be scientifically analyzed to predict illnesses. The analysis of international data collected during health examinations and health status monitoring, along with data collected during medical care delivery, can contribute to precision medicine and the public good. Understanding citizens’ attitudes and predictors of digital health data sharing is critical in promoting data-driven research.

**Objective:**

This study aims to determine the public acceptability of data sharing and the attitudes and influencing factors toward data sharing.

**Methods:**

A cross-sectional web-based survey was conducted in Japan from November 11‐18, 2023. We analyzed 1000 valid responses. Five factors were investigated as predictors of participants’ attitudes toward sharing digital health data for social benefit: (1) individual sociodemographic characteristics, (2) types of health data shared, (3) motivation for sharing data, (4) data sharing concerns, and (5) reasonable access and control over the data. The association of these factors with the respondents’ willingness to share was analyzed. We summarized demographic characteristics based on gender, age group, affiliated educational institution, and education history and degree. Continuous variables are expressed as mean (SE). Logistic regression was used to analyze the association between attitudes and acceptability of sharing digital health data and the predicting factors, such as participants’ preferences regarding data access and control, underlying concerns, motivations for data sharing, demographic characteristics, and eHealth literacy.

**Results:**

The mean age of the participants and the SD was 52.8 (19.8) years. We identified the factors influencing respondents’ willingness to share a wide range of personal digital health data in Japan, including data in medical records, biobank samples, and digitized social communication. Approximately 70% of the participants were willing to share their digital health data. The motives associated with positive willingness to share digital health data were helping future patients (odds ratio [OR] 2.5860, 95% CI 1.8849‐3.5481; *P*<.001), receiving their own results (OR 2.2261, 95% CI 1.6243‐3.0509; *P*<.001), and receiving financial benefits (OR 1.8059, 95% CI 1.2630‐2.5822; *P*=.001). Concerns associated with negative willingness to share data were data being used for unethical projects (OR 0.5104, 95% CI 0.5104‐0.722; *P*<.001) and agreeing to contract terms that they did not fully understand (OR 0.7114, 95% CI 0.5228‐0.9681; *P*=.04). Compared with men, women were less willing to share data (OR 0.722, 95% CI 0.539‐0.967; *P*=.03). Furthermore, the higher one’s eHealth literacy, the more positive their willingness to share digital health data (OR 1.0680, 95% CI 1.0450‐1.0920; *P*<.001).

**Conclusions:**

This study found differences in the types of data people are willing to share. Therefore, the significance of sharing data should be fully communicated to people to motivate them to share their data and contribute to their overall health.

## Introduction

The progression of digital technology has enabled the comprehensive analysis of health-related data, facilitating the prediction of potential illnesses [1-3]. The exchange of personal digital health data plays a crucial role in shaping policies that enhance digital health initiatives and in the innovation of new treatment and prevention strategies [4]. Personal health data includes medical records and data from medical examinations [5]. Data from biological samples, such as blood and DNA, are also important types of health data [6]. In addition, family health data, exercise habits, food intake, and lifestyle data are closely related to health [7-9]. The introduction of eHealth records and the development of wearable apps have led to health data digitization [10,11]. Digital health data on heart rate and blood pressure can be collected by mobile devices in several ways. Digital health data, such as social activities, behavior patterns, and location data are useful for the morbidity risk, prevention, and diagnosis of infectious diseases, including COVID-19 and mental health. Other examples of digital health data include travel records made during the COVID-19 pandemic, web-based data on travel locations, and digital health data on social activities [12-14].

Health data can be collected to contribute to the public good when shared, including data collected during health examinations and health status monitoring, as well as data collected during the delivery of medical care. Therefore, biobanks, which collect individual biological samples and use them for public benefit, and databanks, which hold electronic medical records, are being developed [15-18].

As data science develops, it will likely be used for personalized medicine and understanding the social determinants of health. In Japan, the Ministry of Health, Labor, and Welfare has formulated the “Medical Digital Transformation: Vision 2030,” a strategy for promoting medical digital transformation that actively supports the usage of health and medical care data [19]. In Europe, the General Personal Data Protection Regulation governs the use of personal data, including patients’ electronic medical records. This is because informed consent is required for personal data collection and sharing, including secondary use for research purposes.

Digitizing health data and enabling its international sharing are crucial steps in improving health care worldwide. By making medical records accessible across borders, patients can receive accurate diagnoses and appropriate treatments regardless of their location. This is particularly beneficial for travelers and expatriates, as well as people in emergencies, since doctors can quickly access their medical history and provide suitable care without unnecessary delays [20]. In addition to benefiting individual patients, the international sharing of health data plays a significant role in disease prevention and control. By monitoring global health trends, medical institutions and governments can detect outbreaks at an early stage, respond quickly, and implement effective containment strategies. The importance of such efforts was especially evident during the COVID-19 pandemic, where data sharing initiatives helped track the spread of the virus and accelerate vaccine development [21].

The digitization and sharing of health data also benefit medical research. Access to large-scale anonymized datasets allows researchers to develop innovative treatments, improve AI-driven diagnostics, and advance personalized medicine [22]. Moreover, standardized digital records contribute to the efficiency of health care systems by reducing administrative burdens and facilitating better coordination between medical professionals, ultimately leading to improved patient outcomes [23].

Furthermore, sharing health data internationally helps bridge the gap between developed and developing regions, fostering collaboration that promotes equitable health care access and reduces global health disparities [24].

Rare diseases provide a salient example of the benefits of international data sharing. Definitions of rare diseases differ by region: in the United States, a rare disease is defined as one affecting fewer than 200,000 patients nationwide [25]; in Japan, fewer than 50,000 patients nationwide [26]; and in the European Union, conditions affecting fewer than 1 in 2000 people in the general population [27]. Due to the small number of patients in each country, there is limited availability of diagnostic, genetic, and other health data, as well as insufficient specialized knowledge. Consequently, diagnoses may be delayed or missed entirely, hindering the development of effective treatment and prevention strategies. However, international sharing of health data can overcome these limitations by enabling accurate diagnosis and facilitating research on therapies and preventive measures [28].

Despite the many advantages of digitizing and sharing health data, it is essential to address privacy, security, and ethical concerns. Strong data protection measures and international regulations must be in place to ensure that personal health information is shared responsibly and securely [29]. By balancing the benefits of accessibility with the need for confidentiality, the global health care community can create a system that maximizes both innovation and patient protection.

However, digital health data sharing may not be fully understood without examining citizens’ attitudes toward data sharing related to health and medical care [30]. While health data digitization and sharing can facilitate research and create social benefits, public acceptability may be a barrier to its successful implementation [30]. Examining citizens’ attitudes toward and acceptance of sharing digital health data may be useful in formulating policies and guidelines for data management [31]. This can help in the development and implementation of policies to promote medical health research through digital health personal data. Such efforts benefit others through secondary use of large-scale data, drug development, verification of adverse effects, and reflection on health care policy, rather than for personal, individual medical treatment purposes. Existing studies have considered the acceptability of biobanks, eHealth data, and the acceptability of and attitudes toward digital health data [32-35]. Citizens’ attitudes and receptivity to digital health data sharing differ depending on the type of data being shared, and concerns regarding personal data and transparency issues in data sharing may affect data sharing. However, the types of data that can be digitized and contribute to health include not only biobanks and comprehensive medical records but also lifestyle and behavioral habits.

Few studies have been conducted to elucidate the factors that predict willingness to share digital health data. One study considered the attitudes toward the willingness of young people in Brazil and Denmark to register their digital health data [36]. Studies in Australia [37] and Europe [38] have found that citizens prefer sharing health data under government oversight. They express concerns about sharing anonymized health data for clinical practice or research purposes unless clear provisions are made regarding its intended purpose, limitations on use, and access restrictions [36-38]. Broader citizen consultation on data use, suggesting this trend, has largely been overlooked [39]. However, there is growing evidence of public support for health data sharing [40,41]. Studies indicate that citizens in Australia [42], the United Kingdom, and the United States [43] are willing to share data aimed at improving health outcomes. The determinants of this willingness remain ambiguous, with limited research addressing issues specific to the Asian region, including Japan. This region, with its diverse cultures, social backgrounds, and varying levels of digitalization, may present different predictors of willingness to share digital health data. A comprehensive understanding of the factors influencing citizens’ willingness to share health data can help identify challenges and opportunities for establishing data collection systems, thereby improving communication and fostering citizen trust in digital health data.

## Methods

### Study Design and Setting

This cross-sectional study was conducted via a web-based questionnaire survey administered across Japan by Cross Marketing Inc, an internet research firm. At the beginning of the questionnaire, participants were clearly informed that their personal data would be strictly protected and anonymized. Furthermore, they were explicitly instructed to respond to questions about their attitudes and intentions toward data sharing on the premise that any shared data would exclude personally identifiable data and privacy would be guaranteed. This explanation was included to ensure participants understood that the protection of personal data was a fundamental prerequisite for data sharing scenarios described in the questionnaire.

**Figure 1.**
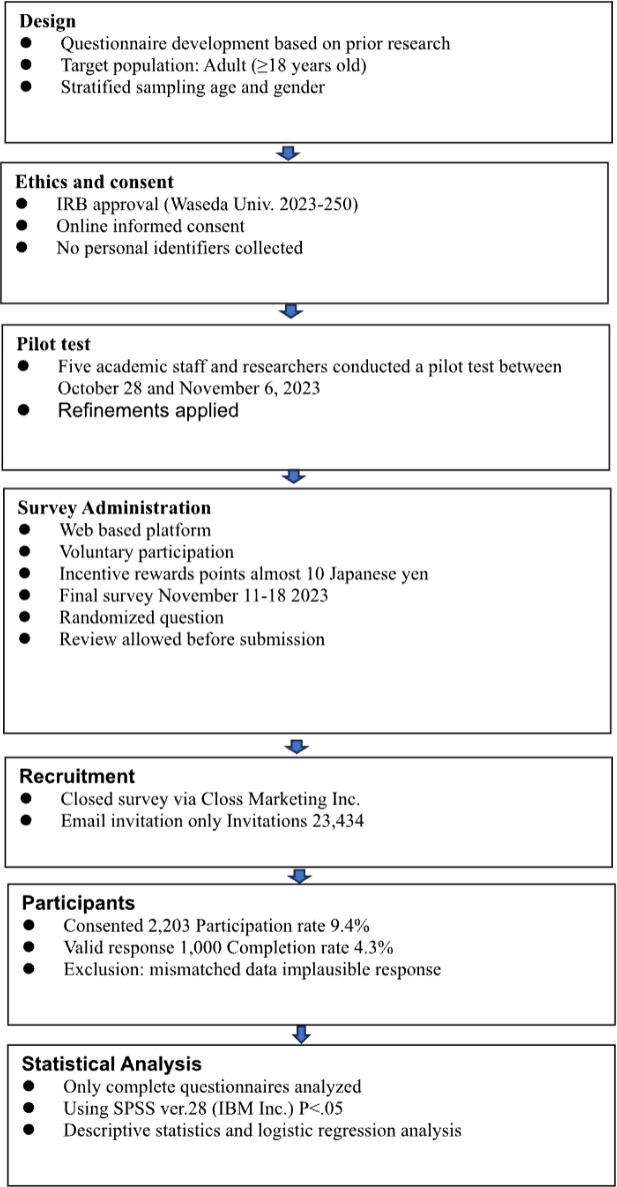
Checklist for Reporting Results of Internet E-Surveys flow chart.

The survey was developed in accordance with the CHERRIES (Checklist for Reporting Results of Internet E-Surveys) checklist (Figure 1, Checklist 1). A pilot test was conducted between October 28 and November 6, 2023, to assess the questionnaire’s clarity, readability, interface usability, and time burden. The final survey was distributed from November 11 to 18, 2023.

### Study Size

A minimum sample size of 385 was calculated using a normal approximation of the binomial distribution, considering Japan’s population of 126 million, a 95% confidence level, and a 5% margin of error [44]. Based on previous studies, 1000 questionnaires were collected to improve the validity of the study [36,38,40].

### Participants and Sampling

Participants were recruited from Cross Marketing Inc.’s panel and were aged 18 years or older. To ensure demographic representativeness, stratified random sampling was used based on age and gender. Participants were equally allocated into seven age categories (18‐27, 28‐37, 38‐47, 48‐57, 58‐67, 68‐77, and 78 years or older) and by gender, resulting in equal numbers in each age category and gender group. Within each stratum, participants were randomly selected.

Exclusion criteria included discrepancies between registered and self-reported gender or age, extremely short response times, failure to follow instructions, or provision of inconsistent or implausible responses. Out of 23,434 distributed questionnaires, 2203 participants provided informed consent, and after applying exclusion criteria, 1000 valid responses were analyzed. Participant demographics are presented in the Results section (Table 1).

**Table 1.** Participant characteristics (N=1000).

Characteristic		Participants, n (%)	
Gender			
Men		500 (50)	
Women		500 (50)	
Age (years)			
18‐27		142 (14.2)	
28‐37		144 (14.4)	
38‐47		144 (14.4)	
48‐57		144 (14.4)	
58‐67		142 (14.2)	
68‐77		142 (14.2)	
>78		142 (14.2)	
Annual household income (Japanese Yen^a^)			
<3 million		196 (19.6)	
3 million–5 million		238 (23.8)	
5 million–8 million		203 (20.3)	
8 million–10 million		92 (9.2)	
>10 million		99 (9.9)	
I don’t want to answer		172 (17.2)	
Education			
Junior high school		32 (3.2)	
High school		285 (28.5)	
Vocational school or junior college		160 (16.0)	
Bachelor’s degree or higher		503 (50.3)	
I don’t want to answer		20 (2.0)	
Marital status			
Unmarried		336 (33.6)	
Married		655 (65.5)	
I don’t want to answer		9 (0.9)	
Owning a mobile phone (smartphone)			
Yes		940 (94.0)	
Owning a personal computer			
Yes		826 (82.6)	
Owning a tablet			
Yes		295 (29.5)	
Owning a smartwatch			
Yes		110 (11.0)	
Owning smart home assistant devices and AI^b^ home appliances			
Yes		65 (6.5)	
Previously participated in health or clinical research			
Yes		57 (5.7)	
Health status			
Bad		27 (2.7)	
Somewhat bad		156 (15.6)	
Neutral		263 (26.3)	
Somewhat good		383 (38.3)	
Very good		168 (16.8)	
Concerned about own and family’s health			
Not concerned at all		24 (2.4)	
Not very concerned		45 (4.5)	
Neutral		108 (10.8)	
Somewhat concerned		400 (40)	
Very concerned		415 (41.5)	
I don’t know		8 (0.8)	

a US$1=147 Japanese Yen (Stand: September 2025).

b AI: artificial intelligence.

### Survey Measures

**Figure 2.**
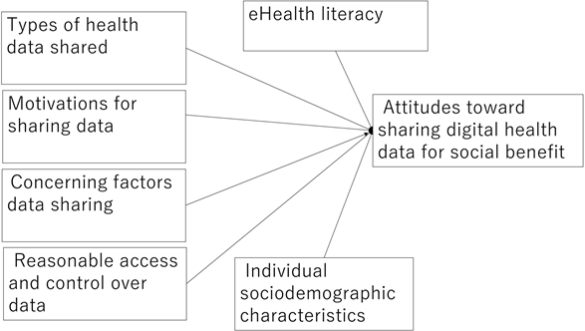
Predictors of public attitude, personal health digital data for social benefit research model.

The questionnaire was developed with reference to previous studies on willingness to share health-related data, including biobank and electronic medical record data, and attitudes toward data repositories. Five factors were investigated as predictors of participants’ attitudes toward sharing digital health data for social benefit: (1) individual sociodemographic characteristics, (2) types of health data shared, (3) motivation for sharing data, (4) concerns factors of data sharing, and (5) reasonable access and control over the data (Figure 2) [36-38,45,45]. A pilot study involving 5 academic staff and researchers helped refine the instrument. The selection and formulation of questionnaire items were based on previous studies identifying factors influencing data sharing behavior.

The questionnaire included the following domains, all using uniform response options (“Yes,” “No,” “Don’t know,” and “Other” with free-text input). Questions were randomized, and multiple responses were allowed.

Full wording of all items and response options is available in Multimedia Appendix 1 questionnaire.

### Willingness to Share Digital Health Data

Participants indicated their willingness to share various types of digital health data, including physical and mental clinical diagnoses, family health history, genetic and blood test data, dietary and nutritional data, alcohol consumption, sleep status, social communication (frequency and content), physical activity, stress, and geolocation data.

### Motivation for Sharing Data

Participants selected motivations such as helping future patients, supporting research, receiving study results, financial incentives, and proposing research topics.

### Concerns About Sharing Digital Health Data

Participants selected concerns from a list including privacy risks, data misuse, unauthorized access, and ambiguity in consent terms. The wording related to access restrictions was revised for clarity. Participants chose from options indicating who may access their data (eg, medical professionals, researchers, and family members) and preferences on data control.

### Attitudes Toward Access Restrictions

Participants reported preferences on controlling access to their data, transparency in data usage, trust in data controllers, and openness to sharing with public or academic institutions.

### Demographics and Health-Related Factors

Data on age, gender, educational attainment, annual household income, marital status, and ownership of digital devices (smartphones, PCs, tablets, smartwatches, and smart home assistants or artificial intelligence [AI] appliances) were collected using predefined categories. Participants also reported previous participation in health research (epidemiological or clinical trials).

eHealth literacy was assessed using the Norman eHealth Literacy Scale (eHEALS), an 8-item, 5-point Likert scale measuring knowledge, comfort, and perceived skills in finding, evaluating, and applying eHealth data [45,46].

Self-rated health status was assessed on a 5-point Likert scale, and responses were grouped into “good” (somewhat good, very good) or “not-so-good” for analysis. Logistic regression analyzed the relationship between health status and willingness to share data.

Participants’ concern about their own and family’s health was measured on a 6-point scale, dichotomized into “interested” or “not interested,” with logistic regression used to assess associations with willingness to share digital health data.

### Statistical Analysis

Descriptive statistics (frequencies, means, and SDs) summarized participant characteristics. We used the intention to share digital health data as the outcome variable. Logistic regression analysis was performed to examine its associations with a range of predictor variables, including motivations for sharing data (eg, helping patients and supporting research), concerns about data sharing (eg, privacy risks and misuse), attitudes toward data access (eg, preferences for control and trust in institutions), and sociodemographic and health-related factors (eg, age, gender, education, income, marital status, digital device ownership, and previous participation in health research). Analyses were conducted using SPSS version 28 (IBM Corp), with significance set at *P*<.05.

### Ethical Considerations

This study fully complied with the Declaration of Helsinki and Japan’s Personal Data Protection Law [47]. Ethical approval was granted by the Waseda University Ethics Committee (application 2023‐250), confirming that all protocols and consent procedures were reviewed and sanctioned before data collection.. No personally identifiable information was collected in accordance with both institutional and legal privacy requirements. All persons described are not identifiable and cannot be identified through the details of the story because this research is an anonymous Internet survey.

Informed consent was obtained electronically before participation. Participants received a small monetary incentive (points equivalent to approximately 10 Japanese yen [US$1=147.1 JPY]) via the survey provider’s point system. Low-quality or inconsistent responses were screened and excluded by the research firm.

## Results

### Characteristics and Attributes of Participants

Table 1 shows participant characteristics. The men-to-women participant ratio is 1:1. The mean age of the participants and the SD is 52.8 (SD 19.8) years. Approximately half of the participants had a university or postgraduate degree in education. In total, 93% of the respondents had a smartphone and 83% had a personal computer. About 80% of the respondents were interested in their health (very interested and somewhat interested).

### Association Between Willingness to Share Digital Health Data and Sociodemographic Characteristics

Approximately 70% of respondents were willing to share digital health data. Respondents who were willing to share digital health data were defined as having a positive willingness to share, and subsequent analyses were conducted.

The results of the logistic analysis of the association between positive attitudes toward sharing digital health data and sociodemographic characteristics are shown in Table 2. Compared with men, women were less willing to share data (odds Ratio [OR] 0.722, 95% CI 0.539‐0.967; *P*<.05).

Those who had more experience participating in research (OR 3.308, 95% CI 1.391‐7.868; *P*<.01), and had awareness of research data repositories (OR 2.711, 95% CI 1.364‐5.388; *P*<.01) were more willing to share digital health data. Moreover, owning a smartphone (OR 1.841, 95% CI 1.078‐3.145; *P*<.05), personal computer (OR 1.658, 95% CI 1.175‐2.341; *P*<.05), tablet (OR 1.688, 95% CI 1.212‐2.349; *P*<.01), or smart home assistance appliances (OR 3.384, 95% CI 1.077‐5.276; *P*<.05) was positively associated with higher willingness to share digital health data. Conversely, there was no association between annual income or education and the willingness to share digital health data. Logistic analysis of e-health literacy and digital health data sharing suggested that the higher the e-health literacy, the more positive the willingness to share digital health data.

Willingness to share digital health data was significantly associated with a high level of health concern and willingness to share digital health data (OR 2.1871, 95% CI 1.5750‐3.03710; *P*<.001) but not associated with health status (*P*=.73).

**Table 2.** Logistic regression results regarding a positive willingness to share personal data and sociodemographic characteristics.

Sociodemographic characteristics	B (partial regression coefficient)	SE	*P* value	OR^a^ (95% CI)
Gender (reference: man)				
Woman	-0.3259	0.1490	.03^b^	0.7219 (0.5391-0.9667)
Age (reference 18‐27 years)				
28‐37	0.3346	0.2679	.21	1.3974 (0.8265-2.3625)
38‐47	0.4610	0.2737	.19	1.5856 (0.9272-2.7115)
48‐57	0.2681	0.2663	.31	1.3075 (0.7758-2.2035)
58‐67	-0.1268	0.2612	.63	0.8809 (0.5280-1.4698)
68‐77	0.1563	0.2697	.57	1.1692 (0.6892-1.9835)
>78	0.0073	0.2628	.98	1.0074 (0.6019-1.6860)
Annual household income (reference:<3 million Japanese Yen^c^)				
3 million–5 million	0.2598	0.2162	.23	1.2967 (0.8487-1.9811)
5 million–8 million	0.3942	0.2341	.10	1.4833 (0.9375-2.3466)
8 million–10 million	0.2782	0.2945	.35	1.3207 (0.7416-2.3521)
>10 million	0.3591	0.2982	.23	1.4320 (0.7982-2.5689)
I don’t want to answer	-0.4969	0.2273	.03	0.6084 (0.3897-0.9499)
Education (reference: secondary school or less)				
High school	-0.4195	0.4269	.33	0.6574 (0.2848-1.5176)
Junior college or vocational school	0.1698	0.4512	.71	1.1851 (0.4895-2.8694)
University or postgraduate	-0.0057	0.4219	.99	0.9943 (0.4350-2.2732)
I don’t want to answer	-0.3880	0.6364	.55	0.6784 (0.1949-2.3618)
Owning digital devices (reference: not owned)				
Mobile phone (smartphone)	0.6104	0.2732	.03^b^	1.8412 (1.0778-3.1453)
Personal computer	0.5058	0.1759	.004^d^	1.6582 (1.1747-2.3408)
Tablet	0.5233	0.1688	.002^d^	1.6876 (1.2123-2.3492)
Smartwatch	0.1524	0.2688	.57	1.1647 (0.6877-1.9724)
Smart home assistant devices and AI^e^ appliances	0.8686	0.4054	.05^b^	2.3837 (1.0770-5.2758)
Knowledge of health databases and experience participating in health research				
Knowledge of research data repositories (reference: no)	0.9972	0.3505	.005^d^	2.7106 (1.3638-5.3876)
Experience participating in health research (reference: no)	1.1964	0.4420	.007^d^	3.3082 (1.3910-7.8680)
eHealth literacy	0.0660	0.0110	<.0001^d^	1.0680 (1.0450-1.0920)
Health status (reference: poor)	0.0478	0.1380	.73	1.0490 (0.8002-1.3750)
Concern about health (reference: no)	0.2840	0.070	<.0001^d^	1.3290 (1.1600-1.5230)

a OR, odds ratio.

b *P*<.01.

c US$1=147 Japanese Yen (stand: September 2025).

d *P*<.05.

e AI: artificial intelligence.

### Willingness to Share Digital Health Data and Types of Data That Can Be Shared

The percentage of respondents who indicated a willingness to share shareable data (multiple responses) is shown in Multimedia Appendix 2 Figure S2. The results show that 44%, 33%, 33%, 32%, and 31% of participants were highly motivated to share data regarding physical clinical diagnoses, sleep patterns, blood samples, stress, and food consumption, respectively. Moreover, the content and frequency of social communication tended to be low (11% and 14%, respectively).

### Predictors of Motivation to Share Digital Health Data and Positive Willingness to Share

Multimedia Appendix 3 Figure S3 shows the percentage of respondents who indicated each item as a motivation for sharing digital health data. Helping future patients was the most common motivation for sharing digital health data (59%), followed by receiving one’s results (48%), supporting researchers (39%), and financial gain (31%).

Table 3 shows the results of the logistic analysis of the association between motives and positive willingness to share data. Motives found to be associated with positive motivation to share digital health data were helping future patients (OR 2.586, 95% CI 1.885‐3.548; *P*<.001), receiving one’s results (OR 2.266, 95% CI 1.624‐3.561; *P*<.001), and receiving financial benefits (OR 1.806, 95% CI 1.263‐2.582; *P*=.001).

**Table 3.** Logistic regression results regarding a positive willingness to share personal health data and motives and motivation to share data.

Motivations for sharing data	B (partial regression coefficient)	SE	*P* value	OR^a^ 95% CI		
Helping future patients	0.9501	0.1614	<.01^b^	2.5860 (1.8849-3.5481)		
Supporting researchers	0.2607	0.1784	.14	1.2978 (0.9148-1.8412)		
Receiving one’s own results	0.8003	0.1608	<.001^b^	2.2261 (1.6243-3.0509)		
Learning the results of the research one participated in	0.5416	0.2158	.012^c^	1.7187 (1.1259-2.6236)		
Receiving financial benefits	0.5911	0.1824	.001^b^	1.8059 (1.2630-2.5822		
Suggesting questions for future studies	0.3823	0.3250	.24	1.4656 (0.7751-2.7712)		

a OR: odds ratio.

b *P*<.01.

c *P*<.05.

### Concerns About Sharing Digital Health Data

The percentage of respondents who selected concerns about their digital health data is shown in Multimedia Appendix 4 FIgure S4 . Of the total respondents, 52% cited unknown use of data for the benefit of others as a concern, approximately 39% agreed to terms and conditions they did not fully understand, and approximately 34% said that data sharing would make them vulnerable to cyber attacks.

Table 4 shows the results of the logistic analysis of the relationship between positive intentions toward digital health data and concerns about sharing. The concern found to be associated with a positive willingness to share data was agreeing to contract terms that were not fully understood (OR 0.711, 95% CI 0.525‐0.968; *P*=.03). The results showed that those who felt concerned tended to have lower positive intentions than those who had no concerns about their data being used for unethical projects (OR 0.510, 95% CI 0.361‐0.722; *P*=.001).

**Table 4.** Logistic regression results regarding a positive willingness to share personal health data and concerns about sharing.

Concerning factors data sharing	B (partial regression coefficient)	SE	*P* value	OR^a^ (95% CI)		
Data being used for the benefit of a company or others without one’s knowledge	0.2864	0.1467	.06	1.3316 (0.9989-1.7752)		
Data being used for unethical projects	-0.6726	0.1770	<.001^b^	0.5104 (0.3607-0.7221)		
Agreeing to terms and conditions without complete understanding	-0.3405	0.1572	.03^c^	0.7114 (0.5228-0.9681)		
Exposure to risks, such as cyber-attacks	-0.0345	0.1597	.83	0.9661 (0.7064-1.3213)		
Being asked to provide more data in the future	0.2406	0.1884	.20	1.2721 (0.8794-1.8401)		

a OR: odds ratio.

b *P*<.01.

c *P*<.05.

### Desirable Access Restrictions Related to Sharing Digital Health Data

Multimedia Appendix 5 FIgure S5 shows the percentage of participants’ responses regarding their preferred access to digital health data sharing. Receiving information about projects using their data was the most common response (43%), followed by receiving information about who is using their data (35%) and deciding who has access to what parts of their data (31%). However, 13% stated they would not need to be contacted after providing their data and having it anonymized.

Table 5 shows the results of the logistic analysis between positive intentions and each of the access restriction items regarding data sharing. The access restrictions found to be associated with positive intentions were receiving information about projects using their data (OR 2.313, 95% CI 1.700‐3.147; *P*=.001), giving public or academic institutions access to their data (OR 2.800, 95% CI 1.9248‐4.0734; *P*=.001), and not receiving information after providing their data (OR 1.929, 95% CI 1.292‐3.029; *P*=.004).

**Table 5.** Logistic regression results regarding a positive willingness to share personal data and data access restrictions.

Reasonable access and control over data	B (partial regression coefficient)	SE	*P* value	OR^a^ (95% CI)		
Receiving information about projects using the shared data	0.8386	0.1571	.001^b^	2.3132 (1.7001-3.1474)		
Not receiving any communication after sharing data	0.6570	0.2301	.001^b^	1.9290 (1.2289-3.0282)		
Receiving information about who is using the shared data	0.1932	0.1664	.25	1.2131 (0.8755-1.6811)		
Deciding who has access to different parts of the data	0.0992	0.1728	.57	1.1043 (0.7871-1.5492)		
Data controllers deciding who has access to the data	0.1525	0.1940	.43	1.1648 (0.7964-1.7036)		
Granting data access to public or academic institutions	1.0297	0.1912	.001^b^	2.8001 (1.9248-4.0734)		

a OR: odds ratio.

b *P*<.01.

## Discussion

### Principal Findings

Internationally, secondary use and third-party provision of personal health-related data have become very important for public health, including surveillance of infectious diseases and the development of preventive methods and treatments [48]. Therefore, it is significant to investigate the factors related to data sharing, such as citizens’ attitudes toward, barriers to, and motivations for data sharing. However, research on citizens’ personal health-related data sharing is limited [49].

Our study identifies factors influencing citizens’ willingness to share a wide range of personal digital health data about health in Japan, including data in medical records, biobank samples, and digitized social communication data. This study showed that approximately 70% of Japanese participants were willing to share digital health data about their health. A survey of public attitudes toward digital research registries among Danish and Brazilian citizens showed that 91% of Brazilian and 70% of Danish citizens had a positive attitude toward digital research registries; our study shows a similar willingness as the results from the Danish participants [36]. Our research focuses on whether people are willing to share data, which directly evaluates behavioral intent. This approach is essential as it provides actionable data for organizations or governments to design effective policies. For instance, it can guide the creation of incentives to promote data sharing or enhance privacy protection measures.

While the Danish and Brazilian study asks about how “comfortable” people feel with data sharing, focusing on emotional aspects, our research directly addresses the question of “willingness to share.” This specificity enables deeper insights into both emotional responses and actual behaviors, bridging the gap between attitudes and actions. Our research design, which evaluates the intent to share data, is not limited by cultural or emotional factors. This makes it versatile and applicable across different global contexts. Conducting similar studies in other countries allows us to uncover universal trends and contrasts in data sharing behavior. Our study suggests that helping future patients is an important motivator for sharing health-related digital health data. Furthermore, participants were willing to share their data in the interest of research.

The participants’ high level of interest in their health suggested that gaining knowledge about their health is a motivating factor. Moreover, social benefits, such as benefits to future patients and benefits to oneself, were simultaneously motivating for sharing digital health data. Researcher benefits were not found to be associated with motivation for data sharing. A study of attitudes toward sharing health data among United States and United Kingdom citizens also reported a lower willingness to share data for commercial purposes compared to that for public benefit [43].

Based on our analysis, willingness to share digital health data regarding physical illness diagnoses, sleep patterns, food intake, blood samples, and stress was relatively high, while participants had a lower willingness to share social communication data. This may occur because social communication can be considered personal data that citizens may be hesitant to share due to privacy concerns [50]. However, social communication provides data for research into mental health and infectious disease control; thus, it holds important implications in public health research. Therefore, there is a need to increase public understanding of the importance and significance of digital health data in this area.

Our results suggest that concerns about providing data to unethical projects and agreeing to contract terms that were not fully understood were barriers to the willingness to share digital health data. A study on public attitudes toward the sharing of personal medical records and genomic data in Japan similarly reported concerns about the unauthorized use of data [35]. Although the indicators differ from this study, a link between literacy in the knowledge of genomics, AI, and digital devices, and the sharing of medical records and genomic data has also been noted [35]. Furthermore, trust is important for data sharing [51,52]. Privacy concerns deter data sharing, particularly attitudes toward sharing health data for health policy creation [53].

Aggregating and analyzing multiple types of health data can have significant benefits. We can gain comprehensive insights into health patterns and conditions by combining diverse sources, such as genetic data, clinical records, and lifestyle habits. This approach supports personalized health care, allowing tailored treatments and interventions to address individual needs effectively. Furthermore, the correlation of health data enables predictive analysis, helping forecast potential health issues and promoting preventative measures [54]. Citizens’ attitudes and receptivity to digital health data sharing differ depending on the type of data; however, concerns persist regarding personal data protection and transparency in data sharing [55].

While one measure to remove barriers of concern is restricting access to digital health data, our results showed that receiving information about projects that use data, getting information about data users, and granting data access to public or academic institutions are linked to a willingness to share data. These results may suggest that people avoid providing data to unethical projects and wish to exercise their right to self-determination regarding data. Other studies have also identified the importance of controlling the sharing of digital health data [36,37].

Our analysis suggests that the sociodemographic factors associated with willingness to share digital health data are being a man; owning smartphones, PCs, and smart appliances; having experience in participating in health research; and having high digital health literacy. It is necessary to consider measures for sharing digital health data that consider individual characteristics. The association between willingness to share digital health data and educational background reported by Grande et al [56] was not found in our study.

### Implications for Policy and Practice

This study identifies factors that make Japanese citizens willing and able to share their data for research. These factors may be applicable not only in Japan but also internationally across borders. An increase in data sharing can contribute to research using big data, for example, the development of diagnostic, preventive, and therapeutic methods for rare diseases for which data are scarce, as well as international surveillance for infectious diseases.

The results of our study suggest that there are differences in the types of data that people are willing to share and that they may be less willing to share data that is related to personal data or not related to their health. Therefore, it is possible to fully explain the significance of sharing through communication with people, motivating them to share, and identifying the possible contributions to their health. Concerns about unethical data use projects and consenting before understanding the full terms of the agreement, identified as barriers to data sharing, can be addressed by disclosing information about the research project when requesting digital health data.

Concerns about unethical data usage and misunderstood consent indicate a demand for stronger ethical governance. Data governance frameworks must incorporate feedback mechanisms, such as providing people with updates about the projects that use their data, thereby enhancing perceived control and accountability.

Receiving this data may contribute to increased confidence in data sharing. Furthermore, ensuring that people have the right to control their data is also key to facilitating data sharing.

The study highlights demographic variations in willingness to share data, suggesting that public engagement strategies must be tailored to specific groups. For instance, women, people with lower eHealth literacy, and those without previous research participation may require more targeted education and reassurance regarding privacy and data control mechanisms.

The willingness of the Japanese public to share health data opens opportunities for international data pooling, particularly in the context of rare diseases and emerging infectious diseases, where domestic data may be insufficient for robust research. These findings support the establishment of cross-border data sharing frameworks that are ethical, secure, and culturally sensitive.

The association between device ownership and willingness to share digital health data suggests that disparities in access to digital technologies may translate into inequities in participation in health research. Policymakers should be aware of the digital divide when designing inclusive health data collection frameworks. These findings underscore the importance of policy measures that enhance public trust in digital health data sharing. Efforts to improve data literacy, transparency in data usage, and the ethical oversight of research projects are vital to increasing citizens’ willingness to share their data. Government and academic institutions should consider developing communication strategies that convey how data will be used, who will access it, and the potential social benefits of data sharing.

### Limitations and Future Challenges

Two methodological limitations exist in this study. First, the web-based survey could have created the possibility of sampling bias as well as bias in education levels and ownership of digital devices.

Another limitation relates to our sampling design. Although we used random selection within strata defined by age and gender, this constitutes stratified random sampling rather than simple random sampling. In our analysis, we treated the sample as if drawn via simple random sampling, which may lead to inaccurate estimation of standard errors and affect inferential validity, including the risk of type I error. However, this design was intentionally chosen to ensure adequate representation across demographic subgroups, particularly to explore associations between data sharing intentions and age or gender. Given the total sample size of 1000, we believe the precision of estimates remains acceptable for our exploratory purposes, although results should be interpreted with appropriate caution.

Third, this study was a survey that asked questions about willingness to share digital health data based on future assumptions. However, the study did not explore the perspectives of data providers regarding implementing actual digital health data sharing.

While the quantitative data provides a broad understanding of attitudes, future research should include qualitative approaches, such as interviews or focus groups, to explore the underlying motivations, fears, and expectations surrounding data sharing in greater depth. Therefore, it is important to survey providers during actual data sharing in a future study. Conducting ongoing research into the topic may also be necessary.

### Conclusion

This study identified people’s receptivity and willingness to share personal digital health data about their health and its influencing factors. Approximately 70% of the respondents viewed sharing digital health data about their health positively. The majority of those with a positive view were motivated to share their data by receiving data about themselves as well as helping future patients. Unwillingness to share digital health data about personal health is a barrier to promoting digital sharing and research for societal benefit. Concerns about data being used in unethical research projects and agreeing to contractual terms and conditions that are not understood were suggested to be associated with reduced willingness to share data. It is also important to improve the understanding of the information of users’ institutions, disclosure of information on the projects used, terms of contracts, and access control of data sharing. Furthermore, associations between willingness to share digital health data for public associations with personal characteristics were found regarding gender, interest in health issues, ownership of digital devices, history of participation in research, awareness of digital health databases, and digital health literacy. Therefore, a digital sharing system that considers individual characteristics and provides information regarding the usage of the data could facilitate data sharing.

## Supplementary material

10.2196/64192 Multimedia Appendix 1 Questionnaire.

10.2196/64192 Multimedia Appendix 2 Figure S2 Types of data that can be shared.

10.2196/64192 Multimedia Appendix 3 FIgure S3 Motivation to share digital health data.

10.2196/64192 Multimedia Appendix 4 Figure S4 Concerns about data sharing.

10.2196/64192 Multimedia Appendix 5 Figure S5 Desirable data access restrictions.

10.2196/64192 Checklist 1 Checklist for Reporting Results of Internet E-Surveys (CHERRIES).

## Abbreviations

CHERRIES: Checklist for Reporting Results of Internet E-Surveys
eHEALS: eHealth Literacy Scale
OR: odds ratio
