# Supplementary material for: Public Attitudes and Predictors of Public Awareness of Personal Digital Health Data Sharing for Research: Cross-Sectional Study in Japan
Source: JMIR Hum Factors. 2025 Oct 9;12:e64192. doi: 10.2196/64192 (PMC12510434; doi:10.2196/64192)
Supplement: Multimedia Appendix 1 [file humanfactors-v12-e64192-s001.docx]

**Informed Consent for Participation in a Research Study**

This questionnaire is part of a research study on public attitudes and intentions regarding the sharing of digital health data. Please read the following information carefully before participating.

**Purpose of the Study**
The purpose of this study is to examine the predictors of individuals’ willingness to share digital health information. By better understanding public opinions and preferences, the findings may contribute to the development of new drugs, diagnostic techniques, preventive methods, and health education.

Please answer the questionnaire with the understanding that the sharing of digital health data for research purposes is assumed to occur under strict protection of personal information and anonymity.

**Data Privacy and Protection**
Your personal information will be strictly protected. No personally identifiable data will be collected, and all responses will be anonymized and kept confidential.
Any digital health data considered in this study will be handled under the assumption that it is fully de-identified and used solely for research purposes with appropriate privacy safeguards in place.

**Ethical Compliance**
This study is conducted in accordance with the Declaration of Helsinki, the Ethical Guidelines for Medical and Health Research Involving Human Subjects (Japan), the Act on the Protection of Personal Information, and other applicable domestic and international laws and ethical standards.

**Voluntary Participation**
Your participation is entirely voluntary. You may choose not to answer any specific question or to stop participating at any time without any penalty.
If you choose to withdraw from the survey, any data you have already provided will not be used in the research.

**Consent to Participate**
By continuing with the questionnaire, you confirm that:

- You are 18 years of age or older, and
- You have read and understood the information above and voluntarily agree to participate in this study.

If you have any questions about this study, please contact the principal investigator.
If you agree to participate, please proceed to the questionnaire.

If you have any questions about this study, please contact the principal investigator.

The principal investigator : Yasue FUKUDA

Mail ;yfukuda@suzuka-u.ac.jp

□I agree to participate in this research

□I do not agree.

A　Demographics and Socioeconomics Questions

1．Please indicate your gender.

□Man

□Woman

2．Please indicate your age.

（　　　　）Years old

What is your age category ?

□18–27 years old

□28–37 years old

□38–47 years old

□48–57 years old

□58–67 years old

□68–77 years old

□78+ years old

3．Please let us know where you live.

□Hokkaido

□Aomori Prefecture

□Iwate Prefecture

□Miyagi Prefecture

□Akita Prefecture

□Yamagata Prefecture

□Fukushima Prefecture

□Ibaraki

□Tochigi Prefecture

□Gunma

□Saitama Prefecture

□Chiba

□Tokyo

□Kanagawa

□Niigata

□Toyama

□Ishikawa

□Fukui Prefecture

□Yamanashi Prefecture

□Nagano Prefecture

□Gifu Prefecture

□Shizuoka Prefecture

□Aichi Prefecture

□Mie Prefecture

□Shiga Prefecture

□Kyoto Prefecture

□Osaka Prefecture

□Hyogo Prefecture

□Nara Prefecture

□Wakayama Prefecture

□Tottori Prefecture

□Shimane Prefecture

□Okayama

□Hiroshima Prefecture

□Yamaguchi Prefecture

□Tokushima

□Kagawa

□Ehime Prefecture

□Kochi Prefecture

□Fukuoka Prefecture

□Saga Prefecture

□Nagasaki Prefecture

□Kumamoto Prefecture

□ Oita Prefecture

□Miyazaki Prefecture

□ Kagoshima Prefecture

□Okinawa Prefecture

4. Please let us know your household income.

□Less than 3 million

□More than 3 million but less than 5 million

□More than 5 million but less than 8 million

□8 million or more but less than 10 million

□More than 10 million

□I do not wish to answer

5. Please let us know your highest education level.

□Middle school

□High school

□Vocational school or junior college

□University or graduate school

□ I do not wish to answer this question

6. Please let us know what digital devices you have.

□Cell phone (smart phone)

□PC/computer

□Tablet

□Smart watch

□Smart Home Assistant Device/AI Appliance

□ Other

7. Please let us know your current health status.

□Poor

□Slightly bad

□Can't say either

□Somewhat good

□Very good

□I don't want to say

8. Are you interested in staying healthy and in your own health issues?

□Not at all interested

□Not very interested

□Can't say either way

□Somewhat interested

□Very interested

□I don't know

9. Have you ever participated in health-related research, including health-related epidemiological studies focusing on nutrition, exercise, lifestyle, and clinical trials for drug development. (research study)?

□Yes

□No

10. A research data repository is an online database containing data collected from previous studies.

Do you know about the Research Data Repository?

□Yes, I know of it

□No, I do not

B Attitude to data sharing for secondary use (for research)

Are you willing to share your health-related information as data with third parties for research purposes? The data is stored in a non-personally identifiable manner so that it can be reused for multiple research

1. Willing to share data for health research?

□Yes

□No

2. What health-related data are you willing to share (multiple responses allowed)?

□Illness　Diagnosis (physical)

□Illness　Diagnosis (Mental)

□Family health

□DNA sample

□Food consumption

□Alcohol consumption

□Sleep patterns

□Blood samples

□Physical activity level

□Stress/emotional level

□Frequency of social communication (calls, ICT use)

□Content of social communication (calls, ICT use)

□Distance traveled

□Places visited

□Other
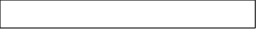


3. please select from the following the motivation/reason for willing data sharing

□Helping future patients

□Supporting researchers

□Receiving one’s own results

□Learning the results of the research one participated in

□Receiving financial benefits

□Suggesting questions for future studies

□Other
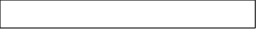


□I don’t know

4. What concerns do you have data sharing?

□Data being used for the benefit of a company or others without one’s knowledge

□Data being used for unethical projects

□Agreeing to terms and conditions without complete understanding

□Exposure to risks, such as cyber-attacks

□Being asked to provide more data in the future

□Other
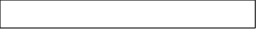


□I don’t know

5. Please select the desired restriction on access to digital data.

□Receiving information about projects using the shared data

□Not receiving any communication after sharing data

□Receiving information about who is using the shared data

□Deciding who has access to different parts of the data

□Data controllers deciding who has access to the data

□Granting data access to public or academic institutions

□Other
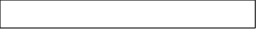


□I don’t know

C Health Literacy

Please select one item that applies to the Internet and medical/health information.

1. I know what health resources are available on the Internet.

□Strongly Disagree

□Disagree

□Undecided

□Agree

□Strongly Agree

２. I know where to find helpful health resources on the Internet.

□Strongly Disagree

□Disagree

□Undecided

□Agree

□Strongly Agree

3. I know how to find helpful health resources on the Internet.

□Strongly Disagree

□Disagree

□Undecided

□Agree

□Strongly Agree

4. I know how to use the Internet to answer my questions about health.

□Strongly Disagree

□Disagree

□Undecided

□Agree

□Strongly Agree

5. Please select "agree" for this question.

□Strongly Disagree

□ Disagree

□ Undecided

□Agree

□Strongly Agree

6. I know how to use the health information I find on the Internet to help me.

□Strongly Disagree

□Disagree

□Undecided

□Agree

□Strongly Agree

7. I have the skills I need to evaluate the health resources I find on the Internet.

□Strongly Disagree

□Disagree

□Undecided

□Agree

□Strongly Agree

8. I can tell high-quality health resources from low-quality health resources on the Internet.

□Strongly Disagree

□Disagree

□Undecided

□Agree

□Strongly Agree

9. I feel confident in using information from the Internet to make health decisions.

□Strongly Disagree

□Disagree

□Undecided

□Agree

□Strongly Agree
