# Supplementary figures and images for: Public Attitudes and Predictors of Public Awareness of Personal Digital Health Data Sharing for Research: Cross-Sectional Study in Japan
Source: JMIR Hum Factors. 2025 Oct 9;12:e64192. doi: 10.2196/64192 (PMC12510434; doi:10.2196/64192)

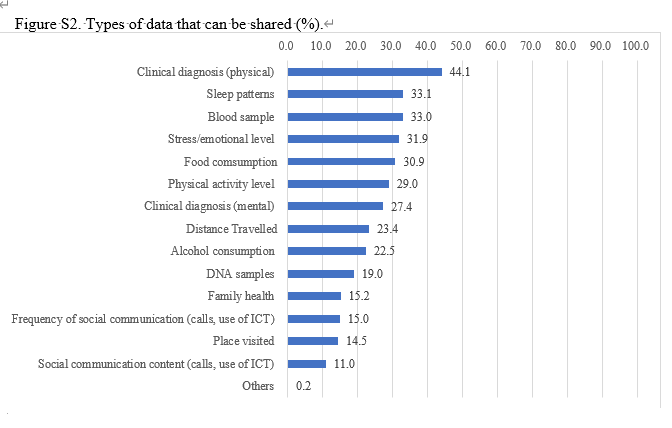

Supplement: Multimedia Appendix 2 [file humanfactors-v12-e64192-s002.png]

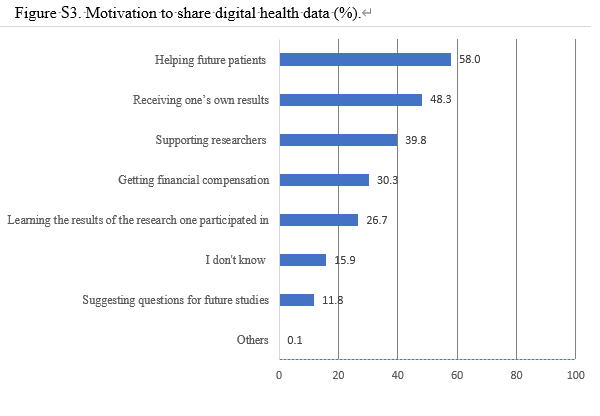

Supplement: Multimedia Appendix 3 [file humanfactors-v12-e64192-s003.png]

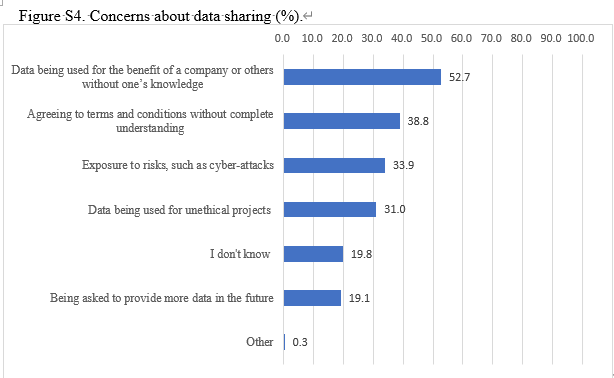

Supplement: Multimedia Appendix 4 [file humanfactors-v12-e64192-s004.png]

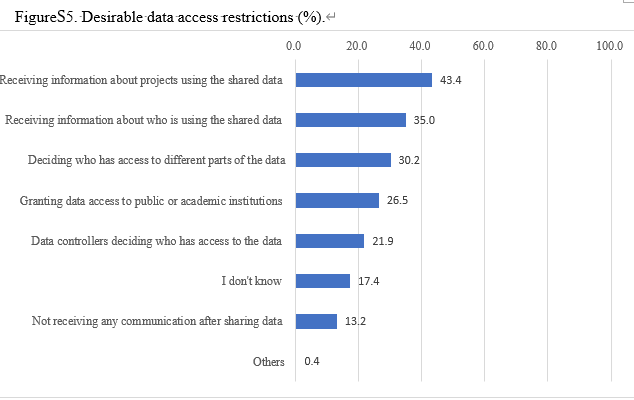

Supplement: Multimedia Appendix 5 [file humanfactors-v12-e64192-s005.png]
