# Supplementary material for: Public Attitudes and Predictors of Public Awareness of Personal Digital Health Data Sharing for Research: Cross-Sectional Study in Japan
Source: JMIR Hum Factors. 2025 Oct 9;12:e64192. doi: 10.2196/64192 (PMC12510434; doi:10.2196/64192)
Supplement: Checklist 1 [file humanfactors-v12-e64192-s006.docx]

# CHERRIES Checklist (Adapted for This Study)

| Item (CHERRIES) | Description in This Study |
| --- | --- |
| 1. Survey design | A cross-sectional web-based questionnaire survey was conducted across Japan. The survey was administered by Cross Marketing Inc., an internet research firm. |
| 2. IRB approval and informed consent process | Ethical approval was granted by the Waseda University Ethics Committee (Application No. 2023-250). At the beginning of the survey, participants were clearly informed that their personal data would be anonymized and strictly protected. They were instructed to answer questions on the premise that no personally identifiable information would be shared and that privacy would be guaranteed. Participation was based on informed consent. |
| 3. Development and pre-testing | The questionnaire was developed with reference to previous studies. A pilot test was conducted from October 28 to November 6, 2023, to evaluate clarity, readability, interface usability, and time burden. Additionally, five academic staff and researchers reviewed the instrument to refine it. |
| 4. Recruitment process and sample description | Participants were recruited from Cross Marketing Inc.’s registered panel. Eligibility: ≥18 years old. Stratified random sampling by age (seven categories) and gender was used to ensure demographic representativeness. |
| 5. Sample size | The minimum sample size was calculated as 385 using a normal approximation of the binomial distribution (Japan’s population: 126 million; 95% confidence level; 5% margin of error). To enhance validity, 1,000 responses were targeted. In total, 23,434 invitations were distributed; 2,203 participants provided consent. After applying exclusion criteria, 1,000 valid responses were retained for analysis. |
| 6. Survey administration | The final survey was conducted from November 11 to 18, 2023. It was self-administered web-based. Questions were randomized, and multiple responses were allowed. Response options were standardized ('Yes,' 'No,' 'Don’t know,' and 'Other' with free-text input). |
| 7. Response rate | Distributed: 23,434 invitations → Consented: 2,203 (9.4% consent rate) → Valid responses: 1,000 (4.3% of distributed). |
| 8. Preventing multiple entries | Cross Marketing Inc.’s panel registration system was used to prevent multiple responses from the same individual. |
| 9. Analysis and exclusion criteria | Exclusion criteria included discrepancies between registered and self-reported gender/age, extremely short response times, failure to follow instructions, or inconsistent/implausible answers. After exclusion, 1,000 responses were analyzed. |
| 10. Survey measures | Survey items were developed based on prior research on willingness to share health data. Five domains were assessed as predictors of attitudes toward sharing digital health data: (1) sociodemographic characteristics, (2) types of health data shared, (3) motivation for sharing data, (4) concerns regarding data sharing, and (5) reasonable access and control over data. The conceptual framework is shown in Figure 1. |
